# Improvement of AlGaN/GaN HEMTs Linearity Using Etched-Fin Gate Structure for Ka Band Applications

**DOI:** 10.3390/mi14050931

**Published:** 2023-04-25

**Authors:** Ming-Wen Lee, Yueh-Chin Lin, Heng-Tung Hsu, Francisco Gamiz, Edward-Yi Chang

**Affiliations:** 1 International College of Semiconductor Technology, National Yang Ming Chiao Tung University, Hsinchu City 30010, Taiwan; ericlmw.st06@nycu.edu.tw (M.-W.L.); nctulin@yahoo.com.tw (Y.-C.L.); hthsu@nctu.edu.tw (H.-T.H.); 2 Department of Electronics and Computer Technology, University of Granada, 18014 Granada, Spain; fgamiz@ugr.es

**Keywords:** AlGaN/GaN HEMTs, etched-fin gate structure, Ka band, linearity, SiC substrate

## Abstract

In this paper, AlGaN/GaN high electron mobility transistors (HEMTs) with etched-fin gate structures fabricated to improve device linearity for Ka-band application are reported. Within the proposed study of planar, one-etched-fin, four-etched-fin, and nine-etched-fin devices, which have 50-μm, 25-μm, 10-μm, and 5-μm partial gate widths, respectively, the four-etched-fin gate AlGaN/GaN HEMT devices have demonstrated optimized device linearity with respect to the extrinsic transconductance ($G_{m}$) value, the output third order intercept point (OIP3), and the third-order intermodulation output power (IMD3) level. The IMD3 is improved by 7 dB at 30 GHz for the 4 × 50 μm HEMT device. The OIP3 is found to reach a maximum value of 36.43 dBm with the four-etched-fin device, which exhibits high potential for the advancement of wireless power amplifier components for Ka band applications.

## 1. Introduction

Over the past decade, the world has seen the rapid spread of transmitting electronic devices in favor of networking and communication systems, such as artificial intelligence (AI), Internet of Things (IoT), and big data. Researchers and industrial engineers have been designing high-speed and high-stability wireless components with III-V semiconductor materials [1,2,3,4]. Therefore, high electron mobility transistors (HEMTs) have now been widely used in high-frequency electronics, such as antennas and broadband satellites [2]. In addition to Gallium Arsenide (GaAs) HEMTs [5,6,7], Gallium Nitride (GaN) HEMTs [8,9,10,11,12] have been used in high radio frequency (RF) power components, such as an mm-wave power amplifier, due to its high breakdown voltage, high critical field, wide bandgap, and high electron peak velocity [13,14,15,16].

At an ideal linear region, an RF HEMT device works as an active device in a Monolithic Microwave Integrated Circuit (MMIC) and could amplify the RF signals with a constant power gain. Nevertheless, the nonlinear characteristics of a realistic solid state HEMT device cause the power gain to decrease after a certain input power, thereby decreasing the device’s output power. Moreover, in a two harmonic wave tone load-pull test, the intermodulation distortion signals (IMD) of the device under test (DUT) increased rapidly with the input power level, which ultimately distorted the fundamental power signal. This is because the input signal of one of the harmonic waveforms intermodulates with the other, and generates third-order intermodulation products, which have now been widely used to quantify the linearity performance of HEMT devices [17,18].

When it comes to improving the linearity of a HEMT device at very high frequency, i.e., the Ka band, the gate controllability performance, such as the $G_{m}$ and $G_{m}$ flatness of the device, becomes the critical factor for the improvement of the device’s linearity characteristics, such as the third-order output intercept point (OIP3) and third-order intermodulation output power (IMD3) [19]. A low second derivative value of the $G_{m}$ curve, meaning a flat $G_{m}$ curve, is favorable for the RF HEMT device to show that the device can withstand the gate voltage swing under high RF input power, keeping the device’s high switching capability as stable as possible [20]. Researchers have shown that better gate controllability could be achieved by etching AlGaN/GaN device gates along the gate width to form fin-shaped gates, overcoming the deficiencies of small gate length GaN devices, which have exhibited poor gate control over the 2DEG channel [21]. The fin-shaped gate structure provides the GaN devices with a high $G_{m}$ value, as well as a flatter $G_{m}$ curve [22,23], which is suitable for high-frequency device operation and serves to mitigate the poor gate control caused by short-channel effects for short-channel AlGaN HEMTs with wide bandgaps [24,25].

However, due to large amounts of etched-away AlGaN barrier layers, AlGaN/GaN FinFETs often suffer from a low saturation current, which makes them unable to provide enough output power for high-frequency data transmission. To increase the RF linearity performance of the AlGaN/GaN HEMT device, as well as maintaining the 2DEG current, the number of etched fins should be limited and optimized.

In this study, AlGaN/GaN HEMTs with different etched-fin gate structures are investigated to improve device linearity for Ka band device applications. The direct current (DC) and RF performance are investigated, and the IMD3 and the OIP3 values are measured to study the linearity improvement of the GaN HEMT device with optimized etched-fin gate structures. The gate controllability as well as linearity performance of the HEMT devices with respect to the different drain biases have also been measured and discussed.

## 2. Materials and Methods

**Figure 1:**
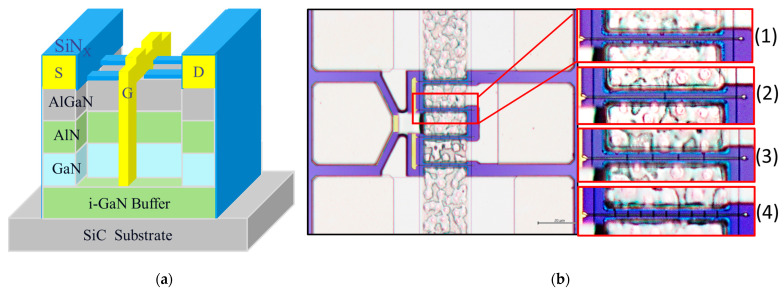
(**a**) The 3D epitaxial and device structure of a 1-etched-fin AlGaN/GaN HEMT device with a single gate, and (**b**) the top-view micrographs of the (1) planar, (2) 1-etched-fin, (3) 4-etched-fin, and (4) 9-etched-fin gate structures.

The AlGaN/GaN HEMTs on a SiC substrate wafer was grown by metal organic vapor deposition (MOCVD) on a 4-inch SiC substrate. From the bottom to the top, the structure of the AlGaN/GaN HEMT consists of a 900 nm i-GaN buffer layer, a 500 nm GaN channel layer, a 1 nm AlN spacer layer, and a 22 nm Al_0.22_Ga_0.78_N barrier layer; the device’s 3D structure is depicted in Figure 1. The room-temperature electron mobility of 1700 cm^2^/V·s and a sheet carrier density of 8.5 × 10^12^/cm^2^ were measured for the structure after material growth.

There are four major steps in the fabrication of AlGaN/GaN HEMTs on a SiC substrate, which include Ohmic contact formation, active region definition, gate formation, and thick metal interconnect fabrication. The Ohmic metal of Ti/Al/Ni/Au was deposited by an e-gun evaporator, and then annealed by a rapid thermal anneal system (RTA) at 850 °C for 30 s in an N_2_ atmosphere. Then, the B^11+^ ion implantation isolation process was used to define the active region. The etched-fin gate region was formed by first depositing the SiN_X_ layer with the plasma-enhanced chemical vapor deposition (PECVD) system and further covering it with an e-beam photoresist.

After this, the etched-fin area was defined using the JEOL e-beam lithography system. The defined fin regions were further etched away using the inductively coupled plasma (ICP) system. In this study, a planar structure (no etched fins) and three different etched-fin gate structures were designed. Trench numbers of 1, 4, and 9 were etched away, with a trench width of 500 nm and a trench depth of around 550 nm to the buffer layer.

Careful removal of the e-beam photoresist after etched fin definition is critical to prevent e-beam photoresist residuals, which may affect the gate length definition during the next e-beam lithography process and may degrade the gate controllability due to poor Schottky contact after gate metal deposition. A larger fin width of up to 500 nm also ensures that the fin etch process is stable and uniform over the whole gate width, which is due to the low aspect ratio of the fin depth and the fin width.

The gate length was defined using the stepper lithography system after the etch fin process, and the wafer was uniformly dipped in a diluted HCl solution (HCl:H_2_O = 1:10) for 1 min before metal deposition to remove any AlGaN barrier layer native oxides. A Ni/Au (50 nm/500 nm) gate Schottky metal was deposited on the defined gate region and was deposited down the etched-fin regions, forming direct contact with the AlGaN barrier layers. Finally, a 150 nm SiN_X_ was passivated on the wafer using the PECVD and a 2 μm thick Au metallization was deposited on the source and drain pads. The schematic cross-section and the top-view micrographs of the device gate structure are shown in Figure 1a,b, respectively, showing the epitaxial material layers and the gate structure with (1) a planar format, (2) 1 etched fin, (3) 4 etched fins, and (4) 9 etched fins.

## 3. Results and Discussion

**Figure 2:**
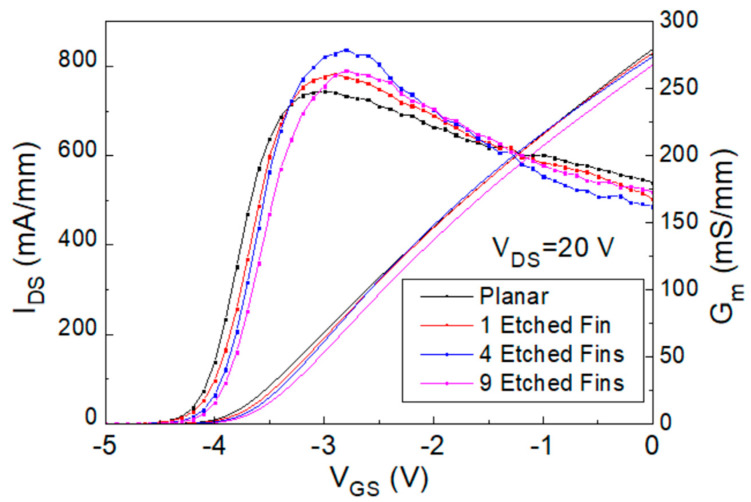
I_DS_-V_GS_ graph of the fabricated planar, 1-etched-fin, 4-etched-fin, and 9-etched-fin AlGaN/GaN HEMTs on a SiC substrate.

Here, 4 × 50 μm AlGaN/GaN HEMTs with different etched-fin gate structures have been fabricated and measured to compare their linearity performance. Figure 2 shows the I_DS_-V_GS_ and G_m_-V_GS_ comparison curves of the fabricated devices with no etched fins (planar), one etched fin (one trench), four etched fins (four trenches), and nine etched fins (nine trenches). The device with four etched fins exhibits the highest $G_{m}$ value and the device with nine etched fins has a highest threshold voltage (V_th_) of −4.05 V. The V_th_ in this study is defined as the V_GS_ when I_DS_ reaches 1 mA/mm. The $G_{m}$ value of the four-etched-fin device increased up to 14% compared to that of the planar device and started to degrade when the etched fin number was increased to nine, which may have been due to the lowering of the gate controllability caused by the increased fin-gate field effect [26], which is also discussed with the Technology Computer-Aided Design (TCAD) simulation results in this study. These transfer characteristics demonstrate the effectiveness of the etched-fin structure in increasing the gate controllability, the device’s gate switching capability, and the potential to withstand voltage and current swinging under high input power RF tests.

**Figure 3:**
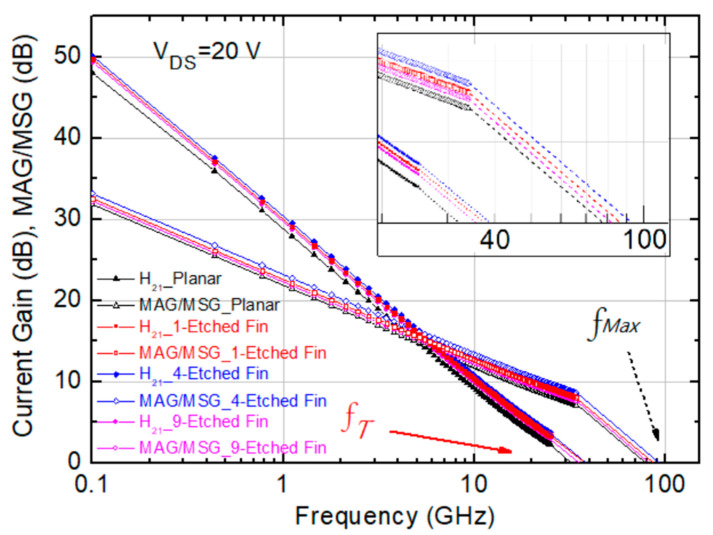
Measured small signal characteristics of the AlGaN/GaN HEMTs on SiC substrate with the planar, 1-etched-fin, 4-etched-fin, and 9-etched-fin gate structures at V_DS_ = 20 V and V_GS_ at G_m_, peak.

Next, S-parameter results were measured on-wafer using the E8361C PNA network analyzer and the 4142B DC supplier. The system was calibrated with a short open load-through calibration standard. The calibration accuracy was verified by ensuring that both S21 and S12 of the through standard were less than ±0.01 dB and that both S11 and S22 were less than −45 dB within the measured frequency range after calibration [27]. The current gain (H_21_) and maximum stable power gain (MSG) were derived using Microwave Office XL, and the f_T_ and f_Max_ of the devices were obtained by extrapolating the gain curves with a slope of −20 dB/decade. Since the current gain versus frequency curves began to deviate from the slope of −20 dB/decade, the gain values above 25 GHz were hidden for clear visualization. The small signal results show obvious improvements with the etched-fin gate structure, and the four-etched-fin device exhibits the highest f_T_ and f_Max_ values of 38.7 GHz and 91.9 GHz among the four device structures at a drain bias of 20 V and a gate bias of −3.05 V, as shown in Figure 3. The f_T_ and f_Max_ of the nine-etched-fin device did not increase with the increased etched fins, which is due to the lowered transconductance and increased gate-to-source capacitance (C_gs_) resulting from the doubled etched fins compared to the four-etched-fin device. The C_gs_ increases with the fin number due to the increased contact area between the gate metal and the semiconductor sidewall, causing the change in f_T_ and f_Max_, as shown in Figure 3.

The polynomial curve fitting technique, using (1), was applied to investigate the I_DS_-V_GS_ curves of the etched-fin devices [18].

(1) $$G_{m}\left(V_{GS}\right)=\frac{\partial I_{DS}{(V}_{GS})}{\partial V_{GS}}=a_{1}+{2a}_{2}V_{GS}+{3a}_{3}V_{GS}^{2}+{4a}_{4}V_{GS}^{3}+{5a}_{5}V_{GS}^{4}+\cdots .$$

Therefore, if we analyze the linearity of the I_DS_-V_GS_ curves, we can see that the I_DS_ increases linearly with V_GS_, giving a lower a_3_ and a_5_, with a larger a_1_ [28]. The I_DS_-V_GS_ polynomial first-, third-, and fifth-order coefficients of V_DS_ = 20 V are listed in Table 1. Decreased $a_{3}$/$a_{1}$ and $a_{5}$/$a_{1}$ values of the devices with four and nine etched fins have been observed, indicating the relatively lower $a_{3}$ and $a_{5}$ values with relatively higher $a_{1}$ values.

For the device’s RF linearity assessment, the OIP3 and IMD3 values could be evaluated using Equations (2) and (3) [18], where $G_{ds}$ is the output conductance, and $R_{L}$ is the load resistance. Since the transconductance characteristics determine the voltage gain of a HEMT device, the influence of the $G_{m}{}^{\prime \prime }$, which is the flatness of the $G_{m}$ curve, on the IMD3 value and the influence of the $G_{m}$ on the OIP3 will be the two main concerns in the following discussion [29].

(2) $$P_{IMD3}\propto \frac{{\left(G_{m}{}^{\prime \prime }\right)}^{2}}{{G_{ds}}^{2}\cdot R_{L}}$$

(3) $$\mathrm{OIP}3\propto \frac{{\left(G_{m}\right)}^{3}}{{{G_{m}{}^{\prime \prime }\cdot G}_{ds}}^{2}\cdot R_{L}}$$

Research has shown that the IMD3 levels of the devices could also be derived as in (4) [18], indicating that the lower $a_{3}$ and $a_{5}$ values could represent lower IMD3 levels.

(4) $$P_{IMD3}=\frac{3}{8}a_{3}A^{3}+\frac{50}{32}a_{5}A^{5}.$$

**Figure 4:**
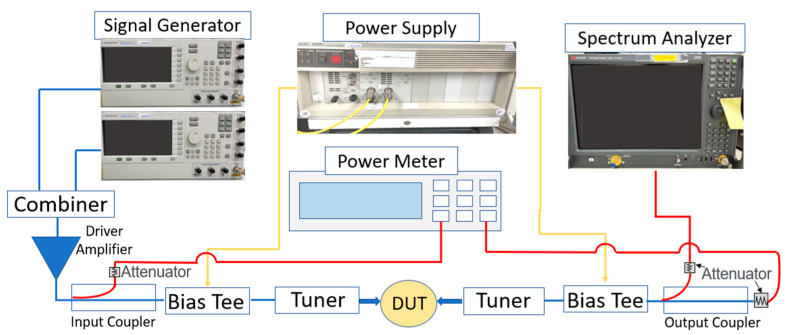
Block diagram of the 2-tone load-pull measurement setup.

To evaluate the device’s RF linearity, two-tone load-pull results were measured with a calibrated 30 GHz frequency signal using the Focus Load-Pull system with a frequency span of 10 MHz. A block diagram of the two-tone load-pull measurement setup with the signal generators, the spectrum analyzer, and the power supply is shown in Figure 4.

**Figure 5:**
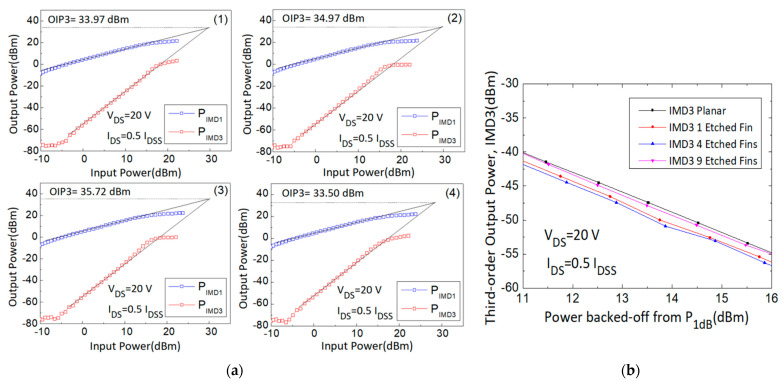
(**a**) Measured output power versus input power curves with the (**1**) planar, (**2**) 1-etched-fin, (**3**) 4-etched-fin, and (**4**) 9-etched-fin gate structures at V_DS_ = 20 V and I_DS_ = 0.5 I_DSS_, and (**b**) IMD3 versus power backed off from P_1dB_ curves of the AlGaN/GaN HEMTs on SiC substrate.

**Figure 6:**
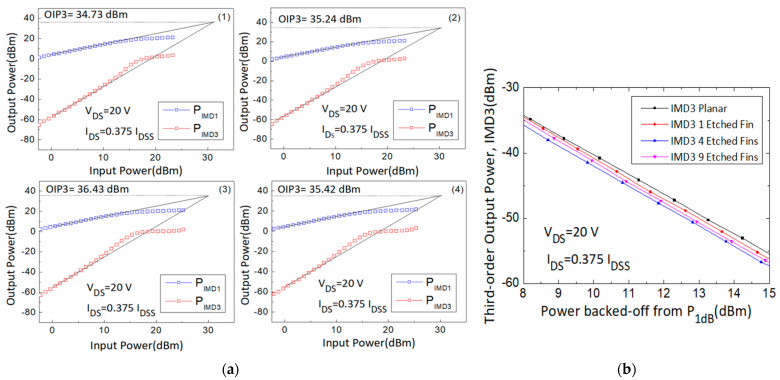
(**a**) Measured output power versus input power curves with the (**1**) planar, (**2**) 1-etched-fin, (**3**) 4-etched-fin, and (**4**) 9-etched-fin gate structures at V_DS_ = 20 V and I_DS_ = 0.375 I_DSS_, and (**b**) IMD3 versus power backed off from P_1dB_ curves of the AlGaN/GaN HEMTs on SiC substrate.

**Figure 7:**
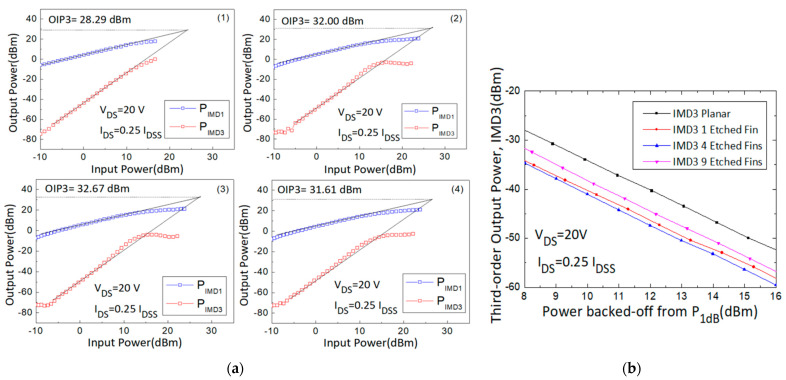
(**a**) Measured output power versus input power curves with the (**1**) planar, (**2**) 1-etched-fin, (**3**) 4-etched-fin, and (**4**) 9-etched-fin gate structures at V_DS_ = 20 V and I_DS_ = 0.25 I_DSS_, and (**b**) IMD3 versus power backed off from P_1dB_ curves of the AlGaN/GaN HEMTs on SiC substrate.

**Figure 8:**
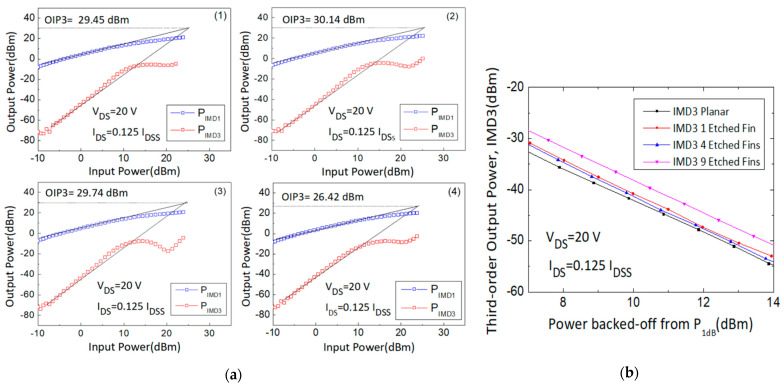
(**a**) Measured output power versus input power curves with the (**1**) planar, (**2**) 1-etched-fin, (**3**) 4-etched-fin, and (**4**) 9-etched-fin gate structures at V_DS_ = 20 V and I_DS_ = 0.125 I_DSS_, and (**b**) IMD3 versus power backed off from P_1dB_ curves of the AlGaN/GaN HEMTs on SiC substrate.

First-order intermodulation output power (IMD1) and IMD3 values were measured and OIP3 values were extrapolated using the fundamental power (F1) and third-order intermodulation power (2F1-F2) data curve with a slope of 1 and 3, respectively, at the linear region. The large signal results of different gate biases (0.5, 0.375, 0.25, and 0.125 I_DSS_) were measured and are shown in Figure 5, Figure 6, Figure 7 and Figure 8.

First, the 30 GHz large signal load-pull measurement results with the IMD3 value comparison results of the designed 4 × 50 μm AlGaN/GaN HEMT devices, biased at I_DS_ = 0.5 I_DSS_ and V_DS_ = 20 V, were analyzed and are shown in Figure 5. The linear gain improved from 7.38 dB to 8.12 dB, the IMD3 level at 16 dB back-off from P1dB (dBm) decreased from −54.82 dBm to −56.72 dBm, the Δ(OIP3-P1dB) value increased from 9.24 dB to 11.26 dB, and the OIP3 value increased from 33.97 dBm to 35.72 dBm. Furthermore, the 4 × 50 μm devices exhibited a maximum power density of more than 2.1 W/mm. The performance of the four different devices is listed in Table 2.

Second, the 30 GHz large signal load-pull measurement results with the IMD3 value comparison results of the 4 × 50 μm AlGaN/GaN HEMT devices, biased at I_DS_ = 0.375 I_DSS_ and V_DS_ = 20 V, were also analyzed and are shown in Figure 6. The linear gain improved from 7.49 dB to 8.38 dB, the IMD3 level at 15 dB back-off from P1dB (dBm) decreased from −55.49 dBm to −57.30 dBm, the Δ(OIP3-P1dB) value increased from 10.60 dB to 12.89 dB, and the OIP3 value increased from 34.73 dBm to 36.43 dBm. The performance of the four different devices is listed in Table 2.

Third, the 30 GHz large signal load-pull measurement results with the IMD3 value comparison results of the 4 × 50 μm AlGaN/GaN HEMT devices, biased at I_DS_ = 0.25 I_DSS_ and V_DS_ = 20 V, were also analyzed and are shown in Figure 7. The linear gain improved from 7.54 dB to 8.28 dB, the IMD3 level at 13 dB back-off from P1dB (dBm) decreased from −52.36 dBm to −59.54 dBm, the Δ(OIP3-P1dB) value increased from 6.73 dB to 11.22 dB, and the OIP3 value increased from 28.29 dBm to 32.67 dBm. The performance of the four different devices is listed in Table 2.

Fourth, the 30 GHz large signal load-pull measurement results with the IMD3 value comparison results of the 4 × 50 μm AlGaN/GaN HEMT devices, biased at I_DS_ = 0.125 I_DSS_ and V_DS_ = 20 V, were also analyzed and are shown in Figure 8. However, although the linear gain improved from 7.39 dB to 7.84 dB, the IMD3 level at 14 dB back-off from P1dB (dBm) increased from −54.73 dBm to −50.83 dBm, the Δ(OIP3-P1dB) value decreased from 11.09 dB to 7.96 dB, and the OIP3 value decreased from 29.45 dBm to 26.42 dBm. The contrasting trends of the results compared to the previous ones show the gate operation voltage limits of these devices. With the $G_{m}$ curves shown in Figure 2, the $G_{m}$ values of the four- and nine-etched-fin devices with I_DS_ = 0.125 I_DSS_ were too low for high-frequency operation, causing the relatively poor OIP3 and IMD3 performance. At I_DS_ = 0.125 I_DSS_, the $G_{m}$ curves of the four- and nine-etched-fin devices showed a larger slope, indicating a large increase in the $G_{m}{}^{\prime \prime }$, and resulting in a larger IMD3 value. The performance of the four different devices is listed in Table 2. The RF results correlate with the trends of the transfer characteristics, and from the measured results, the best operation gate biases were found to lie between 0.5 I_DSS_ and 0.25 I_DSS_ for these etched-fin HEMT devices.

The performance of the four different devices is listed in Table 2, showing the comparison of the RF characteristics with I_DS_ = 0.5, 0.375, 0.25, and 0.125 I_DSS_ at 30 GHz.

From the observations above, the etched-fin device has been concluded to offer obvious improvements regarding the linearity performance compared to that of the planar device, owing to the enhanced gate controllability, represented by the transfer characteristics and right-shifted threshold voltage, which provide the etched-fin devices with a higher power gain under 30 GHz load-pull measurements and improved OIP3 and IMD3 values. However, the nine-etched-fin device has been observed to exhibit lower linearity compared to the four-etched-fin device and one-etched-fin device at specific gate biases. This may be due to the gate electric field effect between adjacent gate fins, and the increase in the C_gs_. With limited numbers of etched fins, the gate controllability could be increased, but when the fin number continues to increase, the fields coming from the gate fins seem to interfere with one another, and this causes the gate controllability to degrade, lowering the $G_{m}$ value and increasing the |$G_{m}{}^{\prime \prime }$| value. Furthermore, the C_gs_ for nine etched fins is higher than in the four-etched-fin device, causing parasitic capacitance effects to deteriorate the device performance, such as the power gain and first and third output power at high-frequency Ka band operation.

To further investigate the buffer deep-etched-fin-gate electric field effect, the AlGaN/GaN HEMT linearity performance with different etched-fin gate structures has been analyzed by changing the drain bias to conduct different drain currents to the channel. The transfer characteristics of the four different devices with different etched-fin gate structures have been measured and two-tone load-pull measurement has been performed at 28 GHz with a frequency span of 10 MHz.

**Figure 9:**
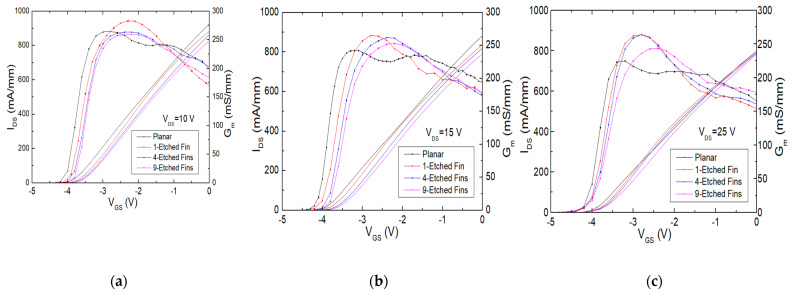
Measured transfer characteristic curves of the AlGaN/GaN HEMTs on SiC substrate with the planar, one-etched-fin, four-etched-fin, and nine-etched-fin gate structures at V_DS_ = (**a**) 10 V, (**b**) 15 V, and (**c**) 25 V.

**Figure 10:**
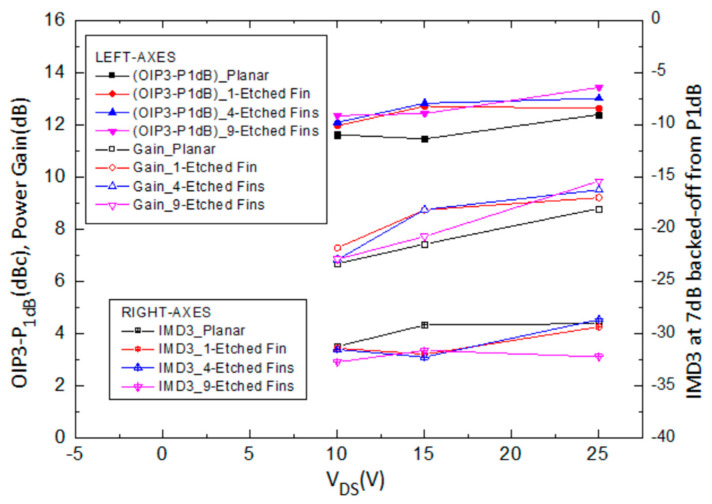
Measured and analyzed Δ(OIP3-P1dB), power gain, and IMD3 level at 7dB back-off from P_1dB_ at different drain biases of the AlGaN/GaN HEMTs on SiC substrate with the planar, 1-etched-fin, 4-etched-fin, and 9-etched-fin gate structures with I_DS_ = 0.25 I_DSS_.

The transfer characteristics of the AlGaN/GaN HEMT devices with different etched-fin numbers, and measured at different drain voltages are shown in Figure 9a–c. At V_DS_ = 10 V, the one-etched-fin device has the highest $G_{m}$ value, while the four- and nine-etched-fin devices have flatter $G_{m}$ curves. The IMD3 shows an improvement with the etched-fin gate design, which is consistent with the transfer characteristic curves at the set operation gate bias for the load-pull measurement, as shown in Figure 9a and Figure 10.

On the other hand, as the drain voltage rises to 15 V and 25 V, as shown in Figure 9b,c, the $G_{m}$ value rises in the one- and four-etched-fin cases, but drops at nine etched fins, which shows that although the existing field effects coming from the gate fins act as a supporter to contribute to the control of the 2DEG channel, there may be a limitation to the number of etched fins, due to the shortened distances between the etched-fin gates, and the decrease in $G_{m}$ may be due to the repelling of charges in the fins [26].

The observations from the transfer characteristics are consistent with those of the RF measurement results. Figure 10 shows the measured and analyzed (OIP3-P1dB), power gain, and IMD3 level at 7 dB back-off from P_1dB_ at different drain biases of the AlGaN/GaN HEMTs on a SiC substrate with the planar, one-etched-fin, four-etched-fin, and nine-etched-fin gate structures. The Δ(OIP3-P1dB) value, power gain value, and IMD3 value versus different drain voltages show that when the devices were operated under lower drain voltages—in this case, 10 V—the devices with four and nine etched fins do not demonstrate significant improvements in power gain and output power, which may be due to the increased gate voltage swing at smaller drain biases [30]. However, at higher drain voltages, the devices show improved performance with increased etched fin numbers, but they still show a limit, which is consistent with the trends shown in Figure 5, Figure 6, Figure 7 and Figure 8.

**Figure 11:**
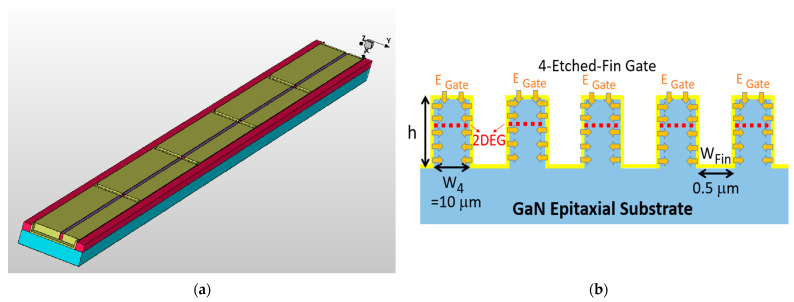
(**a**) The 3D 4-etched-fin AlGaN/GaN HEMT model with a single gate and (**b**) schematic diagram of simulated gate electrostatic potential for the 4-etched-fin device.

**Figure 12:**
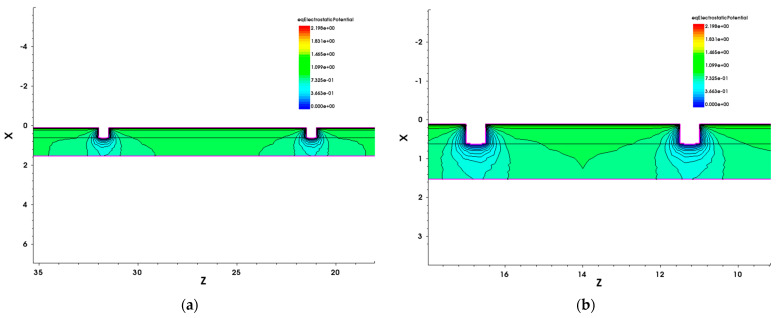
The 3D TCAD simulation results of the electrostatic potential distribution for the etched-fin GaN HEMT with (**a**) 4 etched fins and (**b**) 9 etched fins.

The phenomenon concerning the effects of increased numbers of buffer deep fins has also been analyzed and discussed with the simulation results. The repelling of the electrostatic potential between closely packed gate fins has been modeled and visualized using the Sentaurus TCAD simulation tool. The four-etched-fin three-dimensional (3D) AlGaN/GaN HEMT model was built with a single-etched-fin gate design, as shown in Figure 11a. Figure 11b also shows the schematic diagram of a four-etched-fin device, with the height of the fin (h) and the width of the etched fin (W_Fin_), and the partial width of the gate (W_n_), with n equal to the number of etched fins. W_1_ represents a 25 μm partial gate width, W_4_ is 10 μm, and W_9_ is 5 μm. The cross-sections of the fin gate with the equilibrium electrostatic potential distribution are shown in Figure 12a,b. The distances between the two gate fins in Figure 12a,b are 10 μm (W_4_) and 5 μm (W_9_), respectively. The *X*-axis represents the depth of the etched fin and the *Z*-axis moves along the gate width. The gate voltage is set to −3 V and the drain voltage is set to 20 V. The ranges for the equilibrium electrostatic potential are both set to 0 to 2.19752.

With the schematic diagram in Figure 11b, we can explain that the increase in $G_{m}$ arises from the increase in the effective gate length (L_Gate, eff_). The one-etched-fin device has a L_Gate, eff_ of 4 × h + W_1_, the four-etched-fin device has a L_Gate, eff_ of 10 × h + W_4_, and the nine-etched-fin device has a L_Gate, eff_ of 20 × h + W_9_. However, although the L_Gate, eff_ of the etched-fin gate devices increases with increased etched fin numbers, the results from Figure 12 indicate that the electrostatic potentials between the fin gates of the nine-etched-fin device interact with one another more significantly than in the four-etched-fin device due to the short distances between the sidewall gate. It can be concluded that there exists a repelling of the electric fields from the gate sidewalls, which increases when the etched fins are set to be closer to each other—in this case, W_4_ to W_9_—thus degrading $G_{m}$.

## 4. Conclusions

AlGaN/GaN HEMTs on a SiC substrate with etched-fin gate structures were successfully fabricated and demonstrated good linearity improvements for Ka band applications. The device’s DC and RF performance were improved due to the enhanced gate controllability over the gate width using an optimized etched-fin design. High power gains of more than 8 dB were obtained for the device when operated in a 30 GHz measurement environment. Etched-fin devices show better linearity performance at high frequencies than the planar device due to increased $G_{m}$ values and lowered values of the second derivative of $G_{m}$. The four-etched-fin device, which had an optimized 10-μm separation between the etched fins, exhibited optimized linearity performance under a gate bias point of 0.5 I_DSS_, 0.375 I_DSS_, and 0.25 I_DSS_ among the planar, one-etched-fin, and nine-etched-fin devices at V_DS_ = 20 V. TCAD 3D device simulation results have also been provided to discuss the effect of increased etched fin numbers, which may degrade the gate controllability. Overall, the etched-fin devices demonstrate improved device linearity performance at the Ka band and show high potential for the advancement of wireless power amplifier systems.

## Figures and Tables

**Table 1:** Comparison of the DC characteristics of the four different device structures at V_DS_ = 20 V.

	Planar	1-Etched-Fin	4-Etched-Fin	9-Etched-Fin
I_DSS_ (I_DS_ at V_GS_ = 0 V, mA/mm)	839	830	822	803
G_m, max_ (mS/mm)	247	261	279	264
Threshold Voltage (V)	−4.3	−4.29	−4.20	−4.05
I_DS_-V_GS_ polynomial 1st-order coefficient (a_1_)	−0.12585	0.80071	1.06891	0.36060
I_DS_-V_GS_ polynomial 3rd-order coefficient (a_3_)	−0.07354	0.19897	0.25904	0.06249
a_3_/a_1_	0.58435	0.24849	0.24234	0.17329
I_DS_-V_GS_ polynomial 5th-order coefficient (a_5_)	−0.00137	0.00226	0.00285	0.00023
a_5_/a_1_	0.01089	0.00282	0.00267	0.00064

**Table 2:** Comparison of the RF characteristics of the four different types of devices with I_DS_ = 0.5, 0.375, 0.25, and 0.125 I_DSS_ at 30 GHz.

Device Type (4 × 50 μm)	DC Bias Point: V_DS_ = 20 V, Operation Frequency: 30 GHz						
	RF Bias	I_DS_ (mA)	PIMD3 Level at 16 dB Back-Off from P_1dB_ (dBm)	OIP3 (dBm)	P_1dB_ (dBm)	Δ(OIP3-P_1dB_) (dB)	Gain (dB)
Planar	0.5 I_DSS_	83.90	−54.82	33.97	24.73	9.24	7.38
1-Etched-Fin		83.00	−56.21	34.97	24.13	10.84	7.88
4-Etched-Fin		82.20	−56.72	35.72	24.46	11.26	8.12
9-Etched-Fin		80.30	−55.00	33.50	23.74	9.76	7.79
Planar	0.375 I_DSS_	62.93	−55.49	34.73	24.13	10.60	7.49
1-Etched-Fin		62.25	−56.27	35.24	23.72	11.52	7.84
4-Etched-Fin		61.65	−57.30	36.43	23.54	12.89	8.38
9-Etched-Fin		60.23	−56.86	35.42	23.56	11.86	8.25
Planar	0.25 I_DSS_	41.95	−52.36	28.29	21.56	6.73	7.54
1-Etched-Fin		41.50	−58.17	32.00	21.43	10.57	8.08
4-Etched-Fin		41.10	−59.54	32.67	21.45	11.22	8.28
9-Etched-Fin		40.15	−56.77	30.62	21.02	9.60	7.67
Planar	0.125 I_DSS_	20.98	−54.73	29.45	18.36	11.09	7.39
1-Etched-Fin		20.75	−53.15	30.14	19.24	10.90	7.63
4-Etched-Fin		20.55	−54.11	29.74	18.76	10.98	7.84
9-Etched-Fin		20.08	−50.83	26.42	18.46	7.96	7.00

## Data Availability

Data are contained within the article. The data presented in this study are available in [Study of AlGaN/GaN HEMTs on SiC Substrate with Etched-Fin Gate Structure to Improve Device Linearity for Ka Band Applications].
